# *Arundo donax* L. growth potential under different abiotic stress

**DOI:** 10.1016/j.heliyon.2023.e15521

**Published:** 2023-04-15

**Authors:** Gladys Lino, Paula Espigul, Salvador Nogués, Xavier Serrat

**Affiliations:** a Universitat de Barcelona, Department de Biologia Evolutiva, Ecologia i Ciències Ambientals, Secció de Fisiologia Vegetal., Av. Diagonal 643, 08028, Barcelona, Spain; b Universidad Científica del Sur, Facultad de Ciencias Ambientales, Panamericana Sur Km. 19, 15067, Lima, Peru

**Keywords:** *Arundo donax*, Tolerant, Drought, Salinity, Waterlogging, Temperature, Heavy metals, Phytoremediation

## Abstract

*Arundo donax* L. (giant reed) is a fast-growing, vegetatively multiplying, and rhizomatous perennial grass. It is considered a leading crop for biomass production on marginal and degraded lands under different adverse conditions such as drought, salinity, waterlogging, high and low temperatures, and heavy metal stress. The giant reed tolerance to those stresses is reviewed based on its effects on photosynthetic capacity and biomass production. Possible explanations for the giant reed tolerance against each particular stress were elucidated, as well as changes shown by the plant at a biochemical, physiological and morphological level, that may directly affect its biomass production.

The use of giant reed in other areas of interest such as bioconstruction, phytoremediation, and bioremediation, is also reviewed. *Arundo donax* can be key for circular economy and global warming mitigation.

## Highlights

• *Arundo donax* is a plant species tolerant to diverse types of stress.
• Several tolerance mechanisms help maintain *Arundo donax* production.
• The *Arundo donax* uses encompass other areas, such as bioremediation.
• *Arundo donax* can be essential for circular economy and global warming mitigation.

## 1 Introduction

In the last decade, the European Commission (EC) has been promoting the use of plant's biomass as a biofuel. The latest EC directive on the promotion of the use of energy from renewable sources *(EU 2018/2001)*, established that the member states of the European Union (EU) must gradually increase their biomass consumption at the expanse of fossil fuels, and by 2030, at least 40% of the EU's energy consumption should be provided by renewable resources. This directive will affect many industries, notably the agricultural sector since the introduction of non-food crops for energy production might entail a timely opportunity to uplift incomes and profit margins [1]. In Europe, growing high-yield biomass plants as energy crops on poor and fallow lands can provide a diversified production opportunity for farmers, whilst increasing their profits and additionally reducing financial risks without compromising the food supply [1].

Perennial grasses stand out from others of similar nature for being herbaceous grasses with long perennial life cycles (between 15 and 20 years). Their growth occurs during the warm season resulting in high biomass production and they do not contend for agricultural land as they can be cultivated on marginal or degraded lands [2]. In addition, they provide organic matter which enhance the soil fertility, nutrients retention, structure, and stability of the soil, diminishing soil loss caused through erosion and runoffs [3]. Moreover, their high photosynthetic capacity (ensuring greater CO_2_ fixation), utmost yield potential, significant sugars, lignin and cellulose reservoirs, and low environmental impact (compared to other annual crops), make perennial grasses an attractive source for the production of biofuels [4,5]. In Europe, about 20 perennial species have been studied for biomass production, and among them, three rhizomatous grasses (*Panicum virgatum* L., *Miscanthus* x *giganteus* and *Arundo donax* L.) have been chosen for larger research programs [2]. Among the species previously mentioned, giant reed (*A. donax*) has striking potential for biomass production in the Mediterranean climatic regions [6].

*A. donax* belongs to the Poaceae family and is thought to have spread from Asia to the Mediterranean region (and North Africa) and America [7]. It is one of the largest herbaceous plants in the world. Its canes can reach a height of 8 to 10 m with a diameter of 3 to 4 cm and roots that can reach down to 5 m [8,9]. They have long lanceolate leaves measuring up to 1 m [10]. In the Mediterranean area, giant reed sprouts in early spring (March) spanning its vegetative cycle until flowering between August and November. In late fall, the canes turn yellow and lose their leaves and inflorescences, although the rhizome remains active [10]. *A. donax* has large panicles that contain sterile flowers that produce unviable seeds, being completely agamic. Thus, it somatically reproduces through rhizomes or cane nodes germination, which are dispersed by water flows or by human action. Some authors have considered giant reed an invasive species due to the ease of multiplication [3,11]. Nevertheless, it is not considered an imminent danger due to the absence of sexual reproduction, easing its expansion control. The causes of giant reed infertility are unknown. *A. donax* could be the outcome of a sterile hybrid cross between an *A. plinii* diploid individual and a spontaneous tetraploid of the same species. Another possibility is a cross between an *A. plinii* spontaneous tetraploid and *Phragmites australis* closely related species [12]. Several different chromosomal numbers of *A. donax* have been reported depending on where the plants were sampled (ecotype). The findings range from 24 chromosomes [13] to 122 chromosomes [14]. For this reason, genetic studies in giant reed must be further developed.

Although the giant reed is a C3 metabolism plant, its photosynthetic assimilation, biomass yields, and potential rates are similar to those of C4 species [15]. Despite the growing interest in using perennial grasses as a source of biomass for biofuels, there is still much to learn about basic biology, physiology, biochemistry, genetics, and ecology regarding this species. Hence, it is necessary to study giant reed's physiological and environmental tolerance to identify i) the most suitable ecosystems to maximize its agronomic production [16,17], ii) the best ecotypes adapted to a specific environment, iii) how the crops' age influences in the biomass production, iv) the amount of sugar and starch reserves directly involved in obtaining the final product, and v) the variation in biomass yields according to the soil management and the environmental conditions [18]. Thus, this review aims to compile information about its biomass production under different abiotic stress from the results of various investigations.

## 2 Stress resistance

After the first year of growth, *A. donax* is already stablished thanks to the development of rhizomes and a deep root system [6]. This deep root system makes giant reed tolerant stresses such as flooding, drought, and a vast array of edaphic and climatic conditions. Once this strategy is not enough to maintain the vegetative aerial structures (canes and leaves), the rhizome and root system accumulate sugars and resources to sprout again once the conditions are favorable. For example, according to Nassi et al. [19], at the end of the growing season, the giant reed has a low nutrient content in the aerial biomass, however, the rhizome can provide the necessary support during the spring and storing nutrients in the fall. This feature together with its ability to be maintained for up to 30 years and produce up to 60 t ha^−1^ of dry matter (Table 1), make *A. donax* a suitable alternative for biomass production under adverse environmental conditions.

**Table 1:** Aerial biomass yield (dry matter) and crop age at harvest as reported by authors.

Authors	Topic	Yield (dry matter) (t ha^−1^ y^−1^)	Crop age (years)
Angelini et al. [20]	Biomass yield and energy balance *Unfertilized plants*	17.0	1
		37.0	2
		22.0	3
		18.0	4
		20.0	5
		18.0	6
	Biomass yield and energy balance *Fertilized plants*	19.0	1
		46.0	2
		31.0	3
		28.0	4
		26.0	5
		22.0	6
Cosentino et al. [21]	Evaluation of clones collected in Italy	10.6	1
		22.1	2
Angelini et al. [3]	Long-term field experiment, analysis of productive characteristics	37.1	12
Ceotto et al. [5]	Solar radiation interception and use efficiency	60.0	4
Cosentino et al. [22]	Response to nitrogen fertilization and soil water availability	11.8	2
		16.6	3
		15.9	4
Dragoni [23]	Yield and biomass quality affected by harvest time and frequency	36.5	5
		27.3	6
Proietti et al. [24]	Biomass yield in different experimental places	28.1	6
		14.4	7
		15.5	11

The data shown in this table are approximations based on graphs from published articles.

Some authors have reported an increasing *A. donax* tolerance against any stress under *in vitro*, greenhouse, and field experiments. The main reported giant reed stress tolerance traits are detailed in Table 2.

**Table 2:** Summary of studies on stress tolerance of *Arundo donax* L.

Authors	Country	Stress	Scale	Ecotype	Main results
Mann et al. (2013) [25]	United States of America	Drought Waterlogging	Greenhouse	Californian	100% rhizome fragments viability in waterlogging
Spencer et al. (2013) [26]	United States of America	Waterlogging	Field	Cache Creek Stony Creek	Capable of growing directly in water or on sandbanks
Sánchez et al. (2015) [27]	Spain	Drought Salinity Both	Greenhouse	Piccoplant Martinensis Granadensis Fondachello Tortorici Cefalu Agrigento Licata	The most salinity resistant ecotypes: Fondachello, Licata and Cefalu
Pompeiano et al. (2015) [28]	Italy	Freezing	Greenhouse	Honduran Hungarian	Freeze tolerance ranged from −12.8 °C (Honduran) to −16.4 °C (Hungarian ecotype)
Pompeiano et al. (2015) [29]	Italy	Low oxygen stress	Greenhouse	Local clone	Uses a mechanism characterized by a general restriction in cell metabolism and growth, which allows it to evade stress
Sánchez et al. (2016) [30]	Spain	Drought Salinity Both	Greenhouse	Martinensis	Early stages plants are affected by drought; however, in the same stages they tolerate salinity treatments better
Andreu-Rodríguez et al. (2017) [31]	Spain	Salinity	Field	K-12	High tolerance to saline conditions and a significant adaptation to salt accumulation in soil
Ahrar et al. (2017) [32]	Italy	Drought	Greenhouse	Italian Bulgarian	The Bulgarian ecotype, adapted to drier conditions, exhibits greater drought tolerance than the Italian ecotype
Haworth et al. (2017) [33]	Italy	Drought	Field	Ecotype 6 Ecotype 20	Adaptable to growth in hot and arid Mediterranean climates
Haworth et al. (2017) [34]	Italy	Drought	Greenhouse	Florentine Moroccan	The Moroccan ecotype showed lower photosynthesis, stomatal conductance and photochemical electron transport than the Florentine ecotype
Curt et al. (2018) [35]	Spain	Drought	Field	Attiki Hania Messolonghi Ioannina Torviscosa Rabulese Carcassonne Caltagirone Fondachello	Drought response varied depending on the ecotype. Attiki and Caltagirone were the best ecotypes in biomass production
Romero Munar et al. (2018) [36]	Spain	Drought	Greenhouse	Piccoplant	Great ability to tolerate moderate water stress
Haworth et al. (2018) [37]	Italy	Drought	Greenhouse	Ecotype 6	The stomata respond rapidly to changes in environmental conditions and their behaviour is sensitive to the concentration of ABA in the leaf
Haworth et al. (2019) [38]	Italy	Temperature Drought	Field	Moroccan Sicilian Florentine	Well-adapted in drought-prone areas with warm to hot climates
Riggi et al. (2019) [39]	Italy	Drought	Field	Moroccan Sicilian Florentine	The Moroccan ecotype reported better biomass production under drought than the other ecotypes
Liu et al. [40]	China	Heavy metals	Field	Hunan Yunnan	Great ability to tolerate Cd content up to 525 mg/kg, and Pb content up to 57194 mg/kg. Unaffected growth parameters
Cocozza et al. (2020) [41]	Italy	Salinity/Phosphorus	Greenhouse	Florentine	Prolonged exposure to high phosphorus levels had negative effects of salt stress, but greater activation of physiological mechanisms allowed a prompt and complete recovery after the stress.
Cano et al. (2020) [42]	Spain	Heavy metals	*In vitro*	Ioannina	Showed a broad tolerance to cadmium, chromium, copper, nickel and lead.
Cristaldi et al. (2020) [43]	Italy	Heavy metals	Greenhouse	Fiumefreddo	Good growth capability in polluted soils and a good bioaccumulation capability of heavy metals
Danelli et al. (2021) [44]	Italy	Heavy metals	Field	Ad20	Able to accumulate considerable amounts of Zn and Cu in its harvested biomass
Tshapa et al. (2021) [45]	South Africa	Waterlogging	Greenhouse	Durban	Prefer moist to partially flooded conditions

Similarly, other authors have reported aerial biomass yields from experiments carried out under field conditions. Unfortunately, these field trial studies are still scarce (Table 3).

**Table 3:** Dry aerial biomass production of *A. donax* in field experiments under stress.

Authors	Stress	Ecotype	Dry yield Control (t ha^−1^ y^−1^)	Dry yield Stress (t ha^−1^ y^−1^)	Variation (%)	Crop age (years)
Haworth et al. (2017) [33]	Drought	Ecotype 6	15.0	11.0	−27%	17
		Ecotype 20	10.0	8.5	−15%	17
Andreu-Rodríguez et al. (2017) [31]	Salinity	K12	25.0	22.0	−12%	2
Curt et al. (2018) [35]	Drought	9 ecotypes	43.1	14.7	−66%	2–4
Haworth et al. (2019) [38]	Drought	Morocco	27.5	23.0	−16%	1.5
		Sicilian	19.0	16.0	−16%	1.5
		Tuscan	16.0	15.0	−6%	1.5
Riggi et al. (2019) [39]	Drought	3 ecotypes	23.6	21.0	−11%	1.5
Danelli et al. (2021) [44]	Heavy metals	Ad20	12.5^a^	33.3	+166%	3

The data shown in this table are approximations based on graphs from published articles.

a Control was a mixture of *Medicago sativa* and *Chenopodium album*.

### 2.1 Drought

Drought is one of the top limiting stress factors for plant growth and they are predicted to intensify in the following years. The reduced availability of fresh water and rainfall for irrigation and increased evapotranspiration will have a negative impact on agricultural lands [46]. Plants trigger drought response mechanisms including morphological, physiological, biochemical, cellular, and molecular processes [47,48] such as root system and leaf structure improvement, osmotic and relative water content adjustment, and the fine-tuning regulation of the stomatal opening.

The drought tolerance of giant reed can be explained by the development of solid underground systems with large rhizomes and thick roots that penetrate up to 5 m deep [8,9,19,25]. Thus, it efficiently reaches deep-water sources and accumulates nutrients and carbohydrates [2]. A typical drought adaptation of energy crops is the biomass reduction of the aerial part rather than the underground part [2,25,30,[49], [50], [51], [52]] which does not seriously affect the photosynthetic capacity of giant reed [33]. This indicates that the maintenance of a good root system for the search of underground water sources is more important during water stress conditions [2]. In fact, giant reed is considered a dehydration-prevention species like many other perennial rhizomatous species [2,22,30,36,[52], [53], [54]], since they reduce their stomatal conductance (*g*_*s*_) to avoid water loss and increase their water use efficiency (WUE). Haworth et al. [37] found that giant reed stomata are very sensitive to the first signals of dehydration due to a quick increase in free abscisic acid in the leaf. Simultaneously, it maintains high rates of photosynthesis, which indicates a rapid acclimatization to environmental conditions. Under longer term water scarcity, *A. donax* reduces its foliar biomass while modifies the anatomy of its leaves, making them smaller and thicker [55]. This is a common drought-tolerance mechanism in plants since reducing the size of the leaves reduce the Leaf Area Index and the evaporation of water from the plant to the atmosphere [56,57].

Regarding giant reed field experiments, published results show decreased biomass production in drought-stressed plots, although these reductions were discrete. For example, Haworth et al. [33] reported approximately 15 to 27% biomass reduction in two ecotypes when subjected to drought stress (Table 3). In this experiment, ecotype 6 had the best yield, producing 15 t ha^−1^ y^−1^ under irrigation and 11 t ha^−1^ y^−1^ under water deficit conditions. In a later study using three ecotypes, the trend was similar; reporting between 6 and 16% biomass production decrease when compared to the well-watered controls (Table 3). In this experiment, the Moroccan ecotype had the best performance, 27.5 t ha^−1^ y^−1^ under irrigation and 23 t ha^−1^ y^−1^ under rain-fed conditions [38]. Similarly, Riggi et al. [39] reported an 11% biomass reduction (23.6 t ha^−1^ under irrigation conditions and 21 t ha^−1^ y^−1^ under rain-fed conditions). These results were similar to those presented by Cosentino et al. [21,22], although being lower, in contradistinction to those presented by Angelini et al. [3] and Dragoni [23] (29 t ha^−1^ y^−1^ and 40 t ha^−1^ y^−1^, respectively). These differences in biomass production may be related to four main factors: i) the growth conditions (climatic conditions and soils’ characteristics), ii) the ecotype used in the study, iii) the time between the crop establishment and the stress, and finally, iv) the harvest date [3,33,35,39].

### 2.2 Salinity

Salinity stress severely affects the agrarian production and more than half of the worldwide irrigated agricultural lands are seriously affected by the salinization of their soils [58]. Furthermore, due to the increasing intensity of water stress, the accumulation of salt crusts on agricultural lands has also shot up, especially in the Mediterranean and West Africa regions [59]. In arid and semi-arid environments with high evapotranspiration rates, both water [60] and saline [51] stresses are the most concerning environmental limitations that will negatively affect crops growth and yields in the upcoming future.

In the study carried out by Muller et al. [61], two salinity treatments were tested by adding NaHCO_3_ and Na_2_CO_3_ to the irrigation water at a molar ratio of 1:1 at 80 mM (pH 10.28, 9.1 mS cm^−1^) and 200 mM (pH 10.22, 19.3 mS cm^−1^), which triggered a typical antioxidant response to defend cells against oxidative stress. According to the authors, the response to saline conditions depends on two main factors: i) the rapid activation of the antioxidant defense system and ii) the development of essential strategies to control the transport and accumulation of toxic ions.

Plants show different physiological responses to water and salt stresses which, according to Munns [62], have much in common. Still, these stress tolerance mechanisms are strikingly complex and vary depending on the photosynthesis metabolism, the species, the stress intensity, and exposure time.

In a recent study carried out by Cocozza et al. [41] the response of giant reed to prolonged exposure to salinity (Na), a high concentration of phosphorus (P), and the combination of high levels of Na and P was investigated. According to their results, prolonged exposure to high levels of P worsened the negative effects of salt stress on the photosynthetic performance of plants but enhanced activation of physiological mechanisms that allowed a more fast and complete photosynthesis recovery after stress.

In a field study using glass-fiber polyester containers carried out by Andreu-Rodríguez et al. [31], the giant reed withstood saline conditions up to 5 dS m^−1^, gaining a spot as a halophyte. Significant differences were found in aerial and underground biomass fresh and dry weight when comparing 3 dS m^−1^ (medium stress) and 5 dS m^−1^ (high stress) saline treatments with the non-saline control. Even so, the divergence within both salinity treatments was not compelling. Resultant biomass was 25 t ha^−1^ y^−1^ in salt-free plots, and slightly but significantly lower under salinity stress (Table 3).

In another study carried out by Sanchez et al. [27], eight ecotypes of giant reed got assessed with treatments combining water stress and salinity (WWS-: well-irrigated without salinity, WSS-: water stress without salinity, WWS+: well-irrigated with salinity and WSS+: water stress with salinity). In all ecotypes, values of *A*_*sat*_ (net photosynthesis rate) and *g*_*s*_ decreased. Nevertheless, the study concludes that the giant reed seems to be a salt-resistant plant owing to outright green leaves and shoot/root ratios presented under 0.16 dS m^−1^ salinity (WWS+). In addition, under salinity treatments, the leaves size was reduced and thickened, resulting in lesser water loss [55]. Furthermore, the combined water and salinity stresses treatment (WSS+) affected steeply the growth and development of the plant, which ended up altering the total biomass production. Thus, the giant reed seems more tolerant to salinity than water stress, following the authors’ findings, notably in three out of the eight ecotypes surveyed (Fondachello, Licata and Cefalu). In addition, these same three ecotypes were harvested a few meters above sea level (1, 8 and 16 m. a.s.l., respectively). The rest got gathered between 230 and 773 m. a.s.l.), which may explain their behavior of resistance to saline environments. According to a later Sánchez et al. study [30], the giant reed is more drought-sensitive than salt-sensitive during the establishment of the plant at early stages.

In a recent study, the addition of 5-aminolevulinic acid (ALA), a known promoter of plant growth and abiotic stress tolerance was tested in giant reed under salinity stress, improving its tolerance in preserving the young apical developing leaves from the detrimental effects of salt stress. Thus, the tolerance of *A. donax* to salinity stress can be enhanced by supplying ALA through fertirrigation [63]. Although there are limited reports up to date, they all suggest wide variability in growth, development, and yield responses, depending on the studied giant reed ecotype [21,27,33,35,64].

### 2.3 Waterlogging

The probability of floods will arise, mainly in coastal areas, due to an intensity and frequency increase of extreme rainfall and polar ice melting triggered by global warming [65]. The giant reed is considered an emergent aquatic plant since it can grow directly in water or on sandbanks [26].

Tshapa et al. [45] tested the Durban giant reed ecotype performance under-watered pots allowed to drain (WD), Half Flooded (½F), and 10 cm above the soil level Flooded (F) pots. The ½F treatment worked the best, increasing the total biomass by 80%, the foliar area by 60% and the waterlogging tolerance coefficient by almost 200%. Furthermore, physiological parameters such as CO_2_ uptake, leaf conductance, and quantum yield of photosystem II, were not negatively affected. Additionally, stems were longer and thicker, perhaps owing to greater aerenchyma. This trait is necessary to facilitate long-distance oxygen transport, activating a strategy to avoid anaerobiosis.

Pompeinao et al. [29] studied the behavior of giant reed against oxygen stress (anoxic and hypoxic treatments). The results showed that when subjected to low oxygen level conditions, the plant limits its growth and modifies its metabolism. The expression of the anaerobic *ADH* (alcohol dehydrogenase) gene was firmly increased in leaves and roots under anoxic conditions, while the *ADH* expression under submerged conditions (hypoxic) was relatively low. ADH activity is considered essential for the survival of plants during anaerobic conditions. Giant reed used a mechanism characterized by a general restriction in cell metabolism and growth, which allows evading the anoxia stress. Such a strategy is a typical plant response that regularly withstands deep floods of short duration.

In another experiment by Mann et al. [25], rhizomes of a Californian ecotype got subdued to waterlogging and drought treatments (Control, Flooded, Mild Drought and Extreme Drought). The results showed that the rhizome fragments had 100% viability after flooded and control treatments, while the drought treatments reduced the rhizome viability. On the biomass side, the flooded and control treatments presented the highest results in shoot and root biomass production. Still and all, the drought treatments reduced the production of aerial and underground biomass. Indeed, the metabolic versatility is known to be essential in flooded environments. Furthermore, this waterlogging tolerance of the giant reed makes it suitable for wastewater bioremediation [66,67] and livestock slurry decontamination [68].

### 2.4 Temperature

The average Earth's temperature is considerably increasing due to global warming. Predictions claim that at the current rate of greenhouse gas emissions (GHG) of anthropogenic origin, the temperature will increase 1.5 °C between 2030 and 2052. Plants are vulnerable to abnormal high temperature, especially those displaying C3 photosynthesis and intrinsic low water use efficiency (WUE). This low WUE is a direct consequence of C3 plants high water loss through evapotranspiration [69]. Even though plants are very susceptible to sudden temperature changes, the giant reed has a surprisingly high WUE, similar to C4 photosynthesis plants [15].

Haworth et al. [38], examined three ecotypes: one from a pre-desert area in Morocco, one from a semi-arid Mediterranean climate area in southern Italy, and one from a warm sub-humid region in central Italy were studied. The research concluded the different ecotypes displayed a good plant height, and the number of green and overall leaves growth responded to drought-prone areas with hot to desert climates. However, they failed to fit an ecotype into the “ideotype” concept [70]. They concluded that giant reed is one of the best biomass crops for marginal lands prone to drought and salinity, especially in warm to tropical climates [38].

Besides that, the giant reed is dormant during the late fall and winter periods. It dries out the canes to maintain the underground part (rhizomes and roots) [10]. The giant reed tolerates low temperatures from the first year of establishment. This way, the growth and production of new structures (shoots and rhizomes) in mild winter conditions are portrayed. During the winter dormancy, the giant reed is not very susceptible to frost damage, because of its cold resistance strategy and rhizome resistance protecting its main perennial organ. However, the situation is different when late frosts occur during the spring period [8], since the plant would be subjected to severe physiological damage. In case of prolonged exposure, fatal injuries could occur. Freeze damage is of great importance because winter injuries and late frosts can negatively impact yield, limiting the growth and distribution of the species [71].

Honduran and Hungarian ecotypes, studied by Pompeiano et al. [28] had a cold tolerance of −12.8 °C and −16.4 °C, respectively. They found soluble sugars accumulation in all organs. This feature is common during cold acclimatization, especially in perennial grasses [72,73]. Soluble sugars have an osmotic and non-colligative function in freezing resistance thanks to their low molecular weight, while seem to contribute to the stabilization of membranes [74].

This freezing tolerance advantage will allow the selection of specific cold-tolerant giant reed ecotypes to extend biomass production in a predominantly cold climate and use them for biomass production for bioenergy in the same area.

### 2.5 Heavy metals

Heavy metals soil pollution has become a major global problem for its potential damage to the environment and human health, besides long-term persistence in the environment [75]. Heavy metals enter the human body mainly through dermal contact, inhalation and ingestion [76], which is directly related to agriculture. Heavy metals pollution in agricultural land is an environmental concern [77] since plants grown in contaminated soils can accumulate high heavy metals concentrations. To avoid health risks, the use of non-food crops for bioremediation in contaminated areas is meaningful. Heavy metals pollution is also an issue for aquifers and freshwater quality, extending heavy metals contamination away from the contamination focus. Cadmium (Cd), chromium (Cr), copper (Cu), nickel (Ni) and lead (Pb) are the main heavy metals found in soils.

Phytoremediation seek plant species capable of reducing contaminant levels in a brief span. According to Danelli et al. [44], the giant reed could be a model species for this job since it meets the desired characteristics: i) high biomass production per year and having an economic value, ii) an extensive root system, and iii) good tolerance to heavy metals and accumulation in harvestable biomass.

In an *in vitro* study carried out by Cano-Ruiz et al. [42] with an ecotype named Ioannina, the tolerance to five heavy metals was tested at different concentrations: 0.2, 0.5 and 1 mM CdSO_4_; 0.025, 0.05, 0.1, 0.2 mM K_2_Cr_2_O_7_; 1, 2 mM CuSO_4_ 5H_2_O; 0.1, 0.2, 0.5 NiSO_4_ and 1 mM Pb (NO_3_)_2_. The results showed that the plants did not suffer toxicity symptoms in any treatment except for that of 1 mM Cd, which was lethal for this ecotype. Heavy metals accumulation in the underground part of the plant was stated. This accumulation in biomass is reliant on the dose of heavy metal to which is exposed.

In the study developed by Cristaldi et al. [43], the Fiumefreddo ecotype had an outstanding growth capacity in contaminated soils. At the same time, it presented a high heavy metals bioaccumulation. In addition, Ni, Pb, and Hg increased their bioaccumulation when plants were mycorrhized with *Trichoderma harzianum*.

In another study carried out by Danelli et al. using the Ad20 ecotype [44], the most notable heavy metals present in the harvestable biomass were Zn and Cu. However, the yield was not affected during three years of cultivation; producing 33.3 t ha^−1^ y^−1^ compared to the control (mixture of *Medicago sativa* and *Chenopodium album*) which produced 13.3 t ha^−1^ y^−1^ at the same time (Table 3). In this experiment, *Panicum virgatum* was also evaluated, producing 20 t ha^−1^ y^−1^ of dry matter, with a significant difference compared to giant reed. According to other authors’ publications, these results indicate that *A. donax* is the most productive energy crop in the Mediterranean [3,78,79].

Hunan and Yunnan ecotypes, studied by Liu et al. [40], could survive in intensely polluted soils with a pH between 2.9 and 9.59, a Cd content of up to 525 mg/kg, and a Pb content of up to 57194 mg/kg (non-ferrous mining and smelting area in southern China). At the end of the experiment, the average Cd and Pb content found in the giant reed was 3.32 and 33.8 mg/kg, respectively. In both ecotypes, growth parameters such as leaf area, height, and photosynthetic pigments content were not significantly affected by heavy metals contamination. Furthermore, the cellulose content of the giant reed was not affected in the contaminated soils (average value of 63%), so its biomass could be used as a bioenergy crop while being an excellent option for phytoremediation of large areas of non-ferrous contaminated soil.

Mirza et al. [80] have reported the promising potential of giant reed for the phytoextraction of arsenic (0, 50, 100, 300, 600 and 1000 μg L^−1^) from sewage water. The giant reed plants grew without showing symptoms of toxicity under As concentrations of 50 to 600 μg L^−1^. The potential capacity to serve as a phytoremediation plant lies in the ability to accumulate metals in the stem and leaves above the root concentration. However, the authors reported symptoms of oxidative stress at an As exposure of 1000 μg L^−1^.

In another study conducted by Shaheen [81], giant reed was exposed to different concentrations of Cd (0, 25, 50, 75, and 100 mg/L) to assess its effects on chlorophyll and antioxidant synthesis. The results showed a maximum uptake of Cd (100 mg/L treatment) in the roots (872 mg/kg) followed by the stem (734 mg/kg) and finally the leaves (298 mg/kg). Cd uptake reduced dry weight, chlorophyll *a*, chlorophyll *b*, and total chlorophyll content of giant reed. Despite this, Cd stress was mitigated by giant reed plant cells through the accumulation of antioxidant enzymes and the expression of carotenoid hydroxylases, amidase, glutathione reductase, and transcription factors among others. The authors concluded that giant reed has a great potential for Cd hyperaccumulation based on its robust genetics. This characteristic makes it suitable for phytoremediation purposes.

Phytoremediation is a good alternative to other technologies such as chemical or physical soil remediation [43]. Shortly, phytoremediation strategies will focus on using clean and eco-friendly technologies to restore degraded areas and be able to give them an economic value [82]. Therefore, using plants such as *A. donax* will be of great importance in sectors such as mining and oil extraction to quickly and efficiently remediate the soil pollution.

## 3 Other applications

For many years, giant reed's stems have been used to make musical instruments. In the latter, its resistance envisages the possibility to use it as a building material, especially in sustainable constructions and rural works in places where warm temperatures prevail [83]. Yet in recent years, there has been a revival of its interest as a low-cost, sustainable material source for various construction purposes since it shows good mechanical properties, very similar to those of several bamboo species used in construction, such as *Guadua*, *Phyllostachys violascens* and *Phyllostachys vivax*. Therefore, the giant reed since it is a fast-growing, sustainable material that can be plied for different structures. Other applications in construction areas are wall panels [84], cement mortar reinforced, plywood and particleboard [85]. On the other hand, the leaves are often used as livestock fodder, roofing material, and weaving mats.

The plant can also be used for soil erosion control, as it can grow in degraded lands and has an extensive root system [86]. However, to date, its use for bioenergy production predominates due to its high perennial biomass yield [87] and adverse situations tolerance while requiring little care. In this sector, its use is mainly committed to bioethanol [[88], [89], [90], [91]] and biogas production [92,93].

Giant reed can be used to produce energy through direct combustion but also for 2G biofuels. The 2G biofuels are produced from crops rich in structural carbohydrates, *i.e.* cellulose, hemicellulose or lignin. They produce bioethanol through alcoholic fermentation of lignocellulosic-pretreated biomass, which facilitates sugar release [94]. However, the pretreatment processes used to increase the degradability of carbohydrates consume copious amounts of energy. For this reason, one of the objectives of the genetic improvement of giant reed is the modification of the cell wall composition. Like that, the use of lignocellulose products in biomass is enabled [87].

In plants, polysaccharides are protected in cells by polymers such as lignin. However, in bioethanol production, the presence of high lignin is usually a barrier and can generate inhibitors that hinder hydrolysis and fermentation. That is why a thermochemical pretreatment is usually carried out to eliminate or delocalize the lignin [95,96]. In this sense, an objective to consider for the investigation of biofuels from lignocellulosic crops such as giant reed is to have an efficient pretreatment to eliminate lignin while keeping the cellulose and hemicellulose. However, these pretreatments are expensive and some lead to negative environmental impacts. For this reason, a short-term challenge is to create models of genetically modified plants that present lower levels of lignin, maintaining a high sugar content and conserving their ability to grow on marginal lands [96].

Giant reed could be considered a suitable candidate to complement and even replace corn, sorghum and other 1G bioenergy crops to feed anaerobic digesters and produce green energy. Indeed, its high carbon/nitrogen ratio is needed for animal slurry-based biogas fermenters since animal slurry has low C/N rates for optimum biomethane production [97].

Danelli et al. [98] summarize in four steps the objectives of the genetic improvement of giant reed in the short and medium term: i) plant production (best clones), ii) stand establishment (freeze and herbicide tolerance), iii) crop growth (stress resistance, phytoremediation capacity) and biomass processing (lower lignin content). Studies suggest that clonal selection is the major form of selection for good yield, as well as *in vitro* production and stress exposure experiments, since giant reed being a polyploid species can be difficult to genetically modify.

In recent years the giant reed has become used for the artificial wetlands' construction [[99], [100], [101]], wastewater treatment [66,67,82,101], waste treatment (such as manure derived from farms) [68] and phytoremediation of heavy metal contaminated soils [40,42,43,82,102,103]. According to Fernando et al. [82], the giant reed has considerable future potential for bioremediation as it can improve contaminated water quality, by eliminating the chemical and biological oxygen demand, nitrates, ammonium and phosphate ions, and heavy metals. In the case of contaminated soils (in association with its microorganisms), it can improve the soil properties, both chemically and physically, by removing a good number of heavy metals in contaminated places. At the same time, the plant has good physiological responses and biomass production even under environmentally stressful conditions, obtaining good quality parameters for bioenergy production and other products.

In the current context where the bioeconomy and the green economy have as their main objective the production of biomaterials, energy derived from biomass and renewable energy sources alternative to fossil fuels, giant reed is a relevant option, both for its decontaminating power and its tolerance to various abiotic factors [104]. Therefore, the giant reed can be used for bioremediation and ecosystem services [58], while taking advantage of the biomass for energy production, closing a sustainable economy cycle following the circular economy approach [66,82,98].

## 4 Conclusions

*A. donax* is a plant species tolerant to diverse types of stress without damaging its photosynthetic apparatus, slightly lowering *A*_*sat*_ and *g*_*s*_ levels, while maintaining an acceptable biomass production. In addition, several tolerance mechanisms help maintain the giant reed production, such as extensive roots, the presence of rhizomes, the increase in soluble sugars, the rapid stomatal closure preventing water loss and its increased aerenchyma morphological response.

In recent years, the uses of giant reed are encompassing other areas, such as the bioremediation of contaminated soils, because of the great plasticity of the giant reed to function in different environments. Finally, it can be key to global warming mitigation and the contribution to the circular economy since it can produce renewable and carbon-fixing biomass for bioenergy production while growing on marginal lands or polluted soils.

## Author contribution statement

All authors listed have significantly contributed to the development and the writing of this article.

## Data availability statement

Data included in article/supp. material/referenced in article.

## Declaration of interests

The authors declare the following financial interests/personal relationships which may be considered as potential competing interests: Salvador Nogues reports financial support was provided by University of Barcelona, Spain.
